# Combined Myocardial Bridge and Coronary Vessel Disease Requiring Coronary Artery Bypass Grafting and Myotomy of the Myocardial Bridge

**DOI:** 10.7759/cureus.6486

**Published:** 2019-12-28

**Authors:** Mohammed Al-Musawi, Amanda Marsh, Slee Yi, Suhad AlOmaishi, David Rubay

**Affiliations:** 1 Surgery, Anschutz Medical Campus, University of Colorado, Aurora, USA; 2 Surgery, Charles E. Schmidt College of Medicine, Florida Atlantic University, Boca Raton, USA; 3 Internal Medicine, Life Alliance Organ Recovery Agency, University of Miami, Miami, USA

**Keywords:** myocardial bridging, myotomy

## Abstract

Myocardial bridging (MB) describes a band of myocardium that covers the epicardial surface of the coronary artery. This band can vary both in thickness and distance to which it covers the artery. It is broadly classified as superficial or deep, depending on the thickness of the covering muscle layer. It can be asymptomatic, or it could present with different complications. Reported complications have included ischemia and acute coronary syndromes, coronary spasm, ventricular septal rupture, and arrhythmias. MB is most commonly found in the middle segment of the left anterior descending (LAD) coronary artery. There is controversy with regard to therapy for symptomatic patients who are refractory to medical management. Percutaneous coronary intervention and surgical myotomy (unroofing) have been proposed; yet, each one has its pros and cons. MB can be associated with the development of atherosclerosis proximal to the MB segment in the involved coronary artery, and patients can present having both pathologies. We present a case series of six patients with atherosclerotic coronary lesions requiring coronary artery bypass grafting (CABG) with an accidental perioperative finding of MB, which required myotomy.

## Introduction

Myocardial bridging is a band of myocardium covering the epicardial coronary artery and it has been given different names, such as “myocardial bridge” and “intramural coronary artery” [1]. In 1960, the first angiographic description of myocardial bridges (MB) was reported by Porstmann and Iwig [2]; yet, the majority of them seen at autopsy were not detected angiographically. The incidence of MB ranges from 0.5% to 2.5% in coronary angiographic studies [3], while at autopsy, the incidence was 5% to 86% [4]. Morphologically, MB is classified as superficial or deep, depending on the thickness of the covering muscle layer. Furthermore, the superficial type can be classified as complete or incomplete [4]. Huang et al. examined 37,463 patients with selective coronary angiographic analysis which included 484 with myocardial bridging, a prevalence of 1.3% [5]. Of these 484 patients, 35 received surgical treatment for myocardial bridging with systolic compression of the left anterior descending (LAD) artery. MB is most commonly found in the middle segment of the LAD coronary artery [4, 6].

Traditionally, myocardial bridging has been considered a benign condition; yet, different complications have been reported, such as ischemia and acute coronary syndromes, coronary spasm, ventricular septal rupture, and arrhythmias [7]. There is still controversy with regard to therapy for symptomatic patients who are refractory to medical management, where percutaneous coronary intervention (PCI) with intracoronary stent placement have been proposed as treatments for MBs [8]. However, due to the risk of the in-stent restenosis rate, surgical myotomy (unroofing) offers a complete cure of clinical symptoms and ensures the reversal of local myocardial ischemia which leads to an increase in coronary flow. Nevertheless, surgical myotomy in patients with muscle bridging and a deep coronary artery has the risk of ventricular rupture, aneurysm formation, and postoperative bleeding [7]. LAD artery MBs may significantly differ anatomically with respect to depth (superficial: > 1 to 2 mm vs. deep: > 2 mm) and length of encasement [4, 9].

Pathological studies have clearly shown that MB is associated with the development of atherosclerosis proximal to the MB segment in the coronary artery [10-11]. Recently, the use of advanced multidetector computed tomography (MDCT) has facilitated the non-invasive and accurate assessment of coronary arteries better than conventional angiography. This suggests that MB is a common finding on MDCT and is associated with the development of atherosclerosis proximal to the MB segment [11]. MB should be regarded as an independent risk factor for coronary atherosclerosis and is considered as an important risk factor for coronary heart disease [6]. Having a combined coronary artery lesion and MB is a scenario every cardiac surgeon should expect and be prepared to handle perioperatively. In this study, we are describing the management of cases with combined coronary artery disease (CAD) requiring coronary artery bypass graft (CABG) with the concomitant perioperative finding of MB.

## Technical report

**Figure 1:**
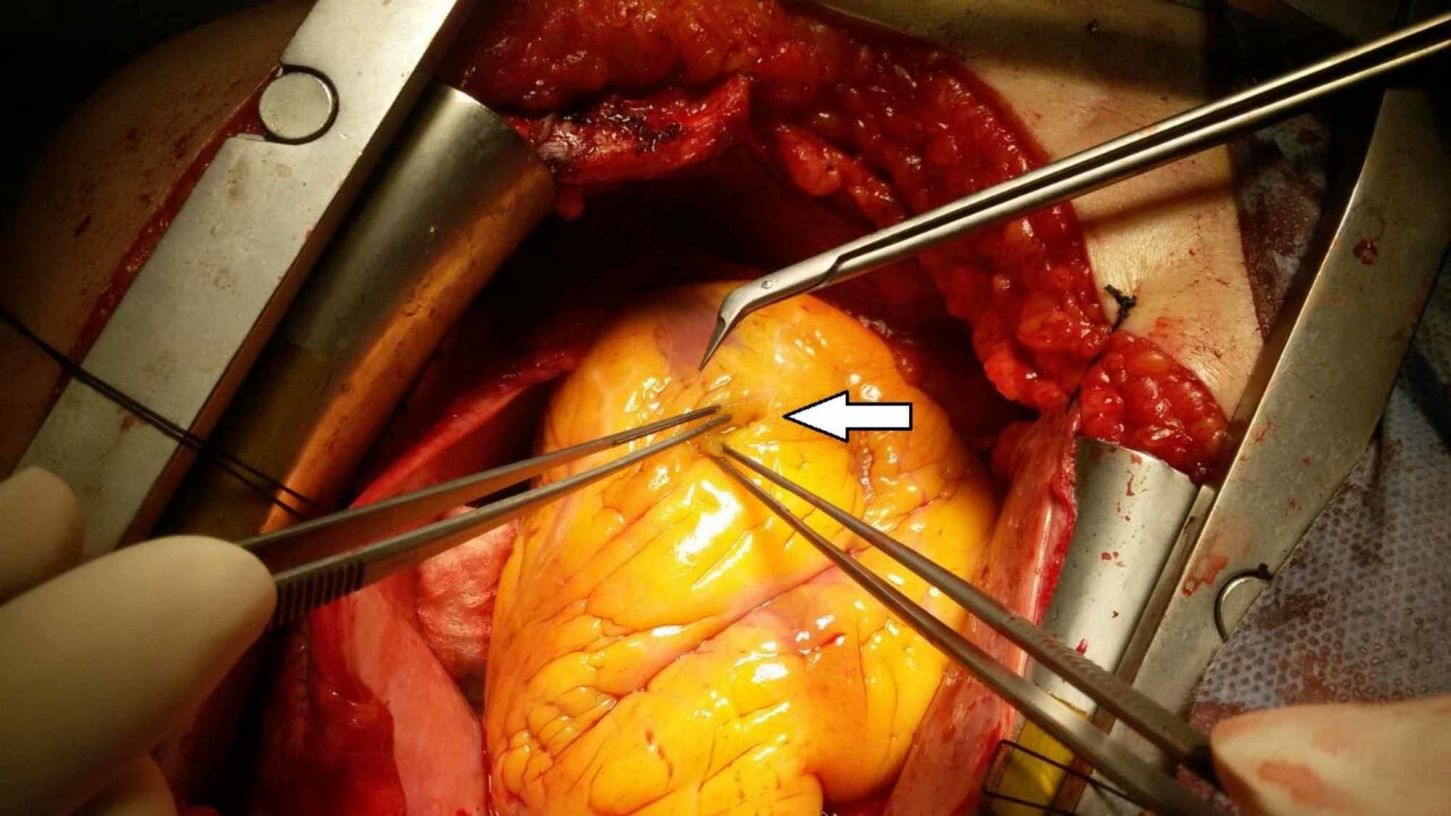
Arrow pointing to the distal end of the left anterior descending (LAD) coronary artery which is totally covered by epicardial fat

**Figure 2:**
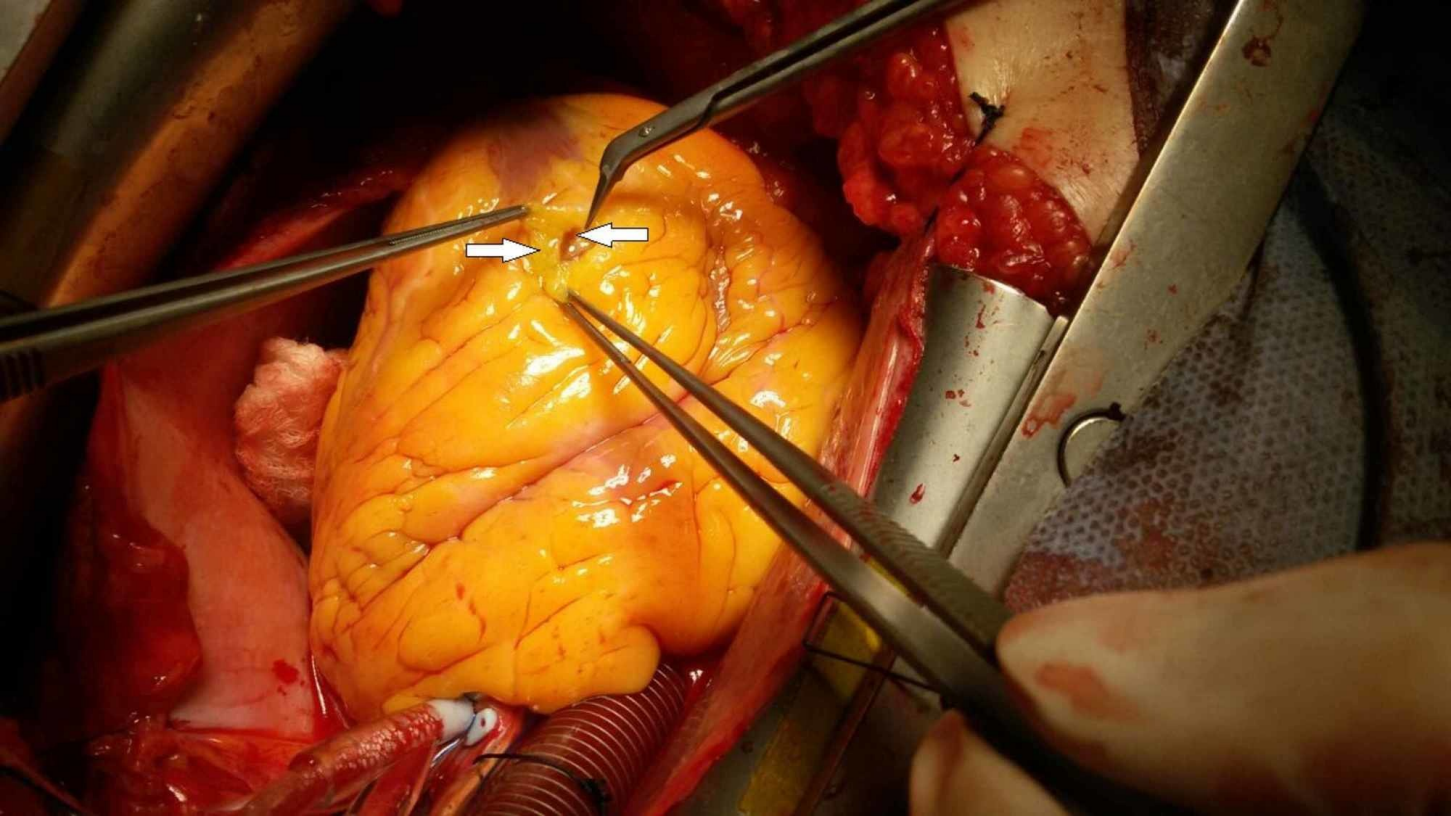
Arrow pointing to the distal end of the left anterior descending (LAD) coronary artery after cutting the overlying epicardial fat and exposing the underlying myocardial bridge (MB)

We describe six cases with MB and coronary arterial ischemic heart disease, for which the patients were referred to surgery to undergo CABG. There was no preoperative diagnosis of MB; all the cases had the diagnosis of MB made perioperatively. When the epicardial fat was dissected, there was an MB covering the LAD coronary artery for variable lengths and thickness, making exposure of the coronary artery distal to the coronary lesion difficult without cutting this band (Figures 1-2). This required retrograde tracking of one of the diagonal branches of the LAD coronary artery until it entered deep below the MB.

**Figure 3:**
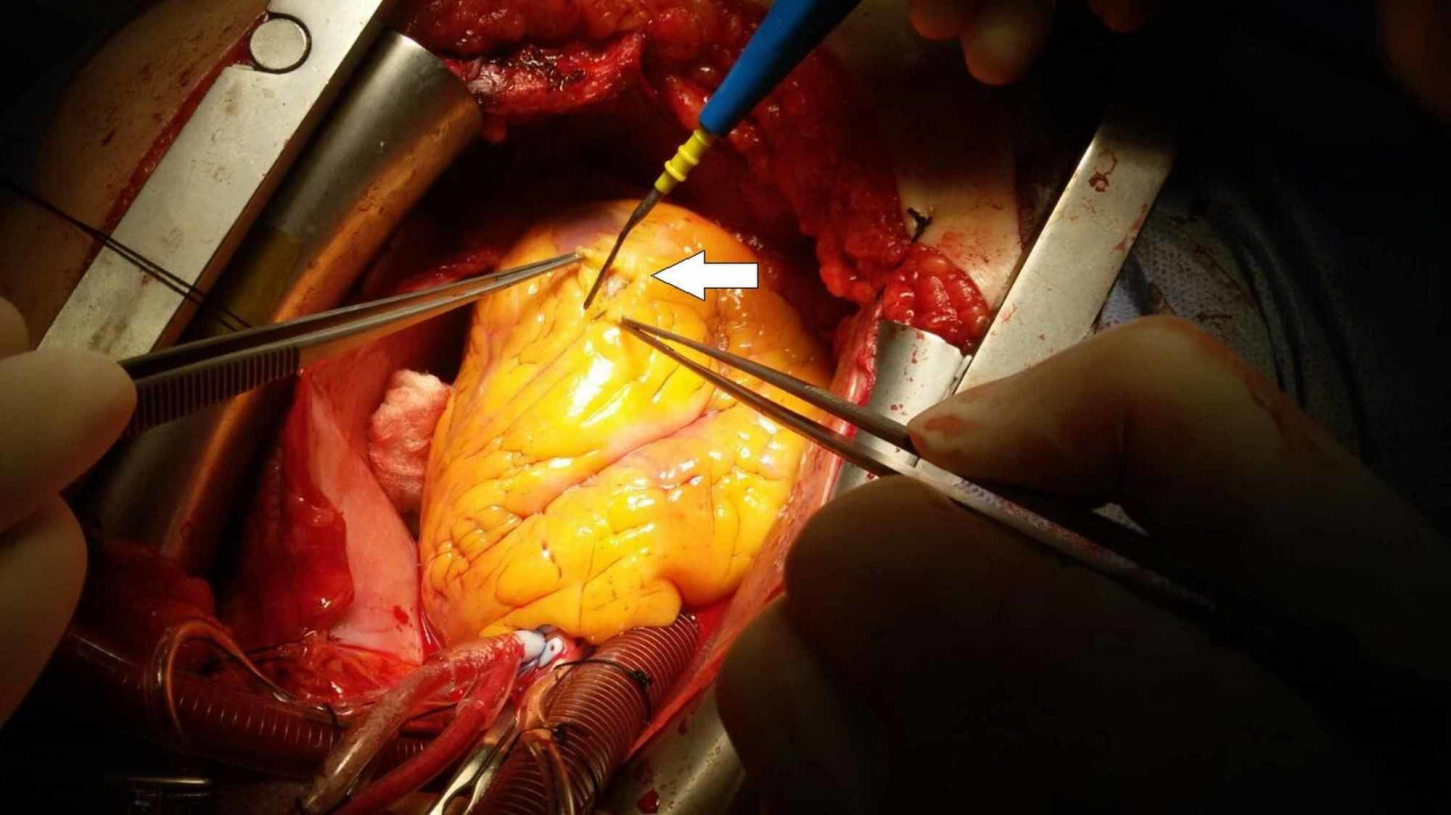
Cutting the myocardial bridge (MB) and exposing the distal part of the left anterior descending (LAD) coronary artery and cauterizing the epicardial fat to expose more of the MB cover of the LAD coronary artery

This approach was taken to avoid inadvertent entry into the LAD coronary artery directly from the MB. From that point, a meticulous dissection of the LAD coronary artery was done. The dissection method was to undermine the MB through the use of a fine-tip hemostat and passing it between the anterior wall of the coronary artery and the undersurface of the MB. After that, we used an electric diathermy with low-energy to cauterize the epicardial fat and the outer part of the MB, providing a hemostatic incision by cauterizing the bridging small epicardial veins (Figure 3).

**Figure 4:**
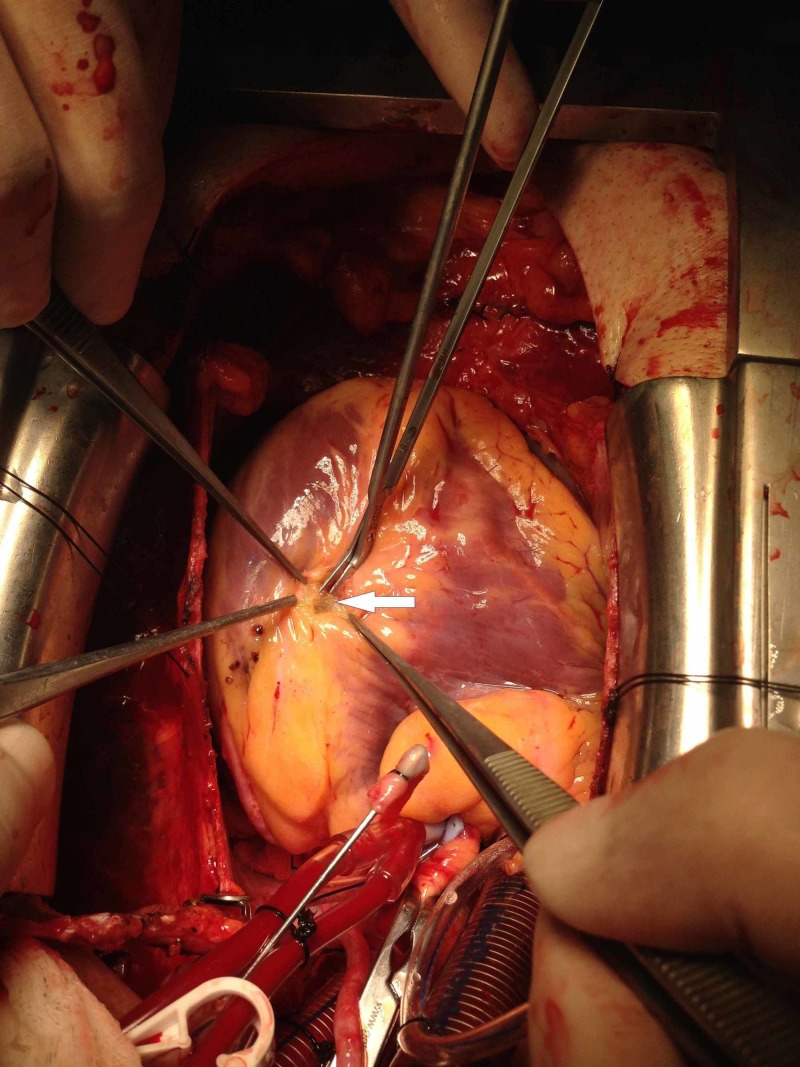
Using fine scissors to cut the myocardial bridge (MB) after having the outer layer of MB cauterized

Thereafter, fine microscissors were used to cut the MB. Care was taken to avoid the inadvertent entry into the right ventricular chamber by directing the dissection more to the left side of the LAD coronary artery (Figure 4).

This process was repeated until we reasonably exposed the LAD coronary artery to allow distal anastomosis to be performed. The dissection required clipping and cauterization of any bridging larger epicardial veins passing through the MB (Figure 3). When the segment of the MB was thick with lots of oozing, we sometimes used an over-and-over eversion suturing technique of the edges of the MB to minimize and/or control significant bleeding, especially in the patients under full heparinization. All the cases had an MB length greater than 3 centimeters, and the thickness ranged from 1 - 4 millimeters. The exposed LAD coronary artery in all cases had very thin arterial walls without any atherosclerotic lesion, apart from the lesion proximal to the MB covered by a layer of fat and myocardium (Figure 4). All six patients had an uneventful postoperative course, except for one who required reexploration due to bleeding from the edges of the MB which were not sutured during the surgery.

## Discussion

Symptomatic MB patients are usually taken care of by the cardiologist rather than a surgeon, and only a few patients with an isolated MB are referred for surgical unroofing or CABG [7, 12-13]. The literature reports that MB is an independent risk factor for developing atherosclerotic coronary arterial lesions and the prevalence of a LAD artery MB is between 23% - 29% [14].

In the six cases presented, none of them had a preoperative diagnosis of MB, and it was a perioperative finding which necessitated a surgical decision. Myotomy was performed in all cases because the MB was covering a long segment of the mid to distal LAD artery. This perioperative anatomical finding made it difficult to find a landing zone immediately distal to the coronary arterial lesion without performing a myotomy.

It is believed that in patients with severe bridging and concomitant CAD who are undergoing CABG, myotomy improves flow to branches proximal to the anastomosis and distal to the coronary arterial lesion [15-16]. Retrograde perfusion of the branches proximal to the distal anastomosis was visually obvious perioperatively while administrating of cardioplegic blood through the graft to the native coronary artery to evaluate flow, patency, and the integrity of the anastomosis. Unfortunately, however, it was not possible to confirm this assumption in our cohort because of the difficulty in determining whether the postoperative improvement in symptoms was due to the CABG alone or the added myotomy. Adding the surgical myotomy to CABG is not without potential risks, including an inadvertent entry to the right ventricle, bleeding from the dissection site, and aneurysmal formation [17]. Bleeding from the edges of the myotomy is another problem that faces the surgeon. To minimize that risk, we followed two techniques depending on the thickness of the MB. We cauterized those with relatively shallow edges, while we sutured the thicker edges using 6/0 Prolene (Ethicon, Inc., Somerville, NJ) to overrun the profuse bleeding branches on both sides of the myotomy. Of all of the cases, we only needed to use both techniques in the case of excessive bleeding.

## Conclusions

Combined coronary artery atherosclerotic disease and myocardial bridging is a surgical pathologic condition which cardiac surgeons may encounter accidentally perioperatively. The role of performing a myotomy is both a necessity to make a landing site for the distal anastomosis and to provide better coronary perfusion to areas distal to the coronary arterial lesion and proximal to the distal coronary anastomosis.

## References

[1] Alegria JR, Herrmann J, Holmes DR Jr, Lerman A, Rihal CS (2005). Myocardial bridging. Eur Heart J.

[2] Porstmann W, Iwig J (1960). Intramural coronary vessels in the angiogram (Article in German). Fortschr Geb Rontgenstr Nuklearmed.

[3] Kramer JR, Kitazume H, Proudfit WL, Sones FM Jr (1982). Clinical significance of isolated coronary bridges: benign and frequent condition involving the left anterior descending artery. Am Heart J.

[4] Tarantini G, Migliore F, Cademartiri F, Fraccaro C, Iliceto S (2016). Left anterior descending artery myocardial bridging: a clinical approach. J Am Coll Cardiol.

[5] Huang X, Wang S, Xu J (2007). Surgical outcome and clinical follow-up in patients with symptomatic myocardial bridging. Chin Med J (Engl).

[6] Nakaura T, Nagayoshi Y, Awai K, Utsunomiya D, Kawano H, Ogawa H, Yamashita Y (2014). Myocardial bridging is associated with coronary atherosclerosis in the segment proximal to the site of bridging. J Cardiol.

[7] Sun X, Chen H, Xia L, Zhao D, Ding W, Wang C (2012). Coronary artery bypass grafting for myocardial bridges of the left anterior descending artery. J Card Surg.

[8] Stables RH, Knight CJ, McNeill JG, Sigwart U (1995). Coronary stenting in the management of myocardial ischaemia caused by muscle bridging. Br Heart J.

[9] Kim PJ, Hur G, Kim SY, Namgung J, Hong SW, Kim YH, Lee WR (2009). Frequency of myocardial bridges and dynamic compression of epicardial coronary arteries: a comparison between computed tomography and invasive coronary angiography. Circulation.

[10] Ge J, Erbel R, Görge G, Haude M, Meyer J (1995). High wall shear stress proximal to myocardial bridging and atherosclerosis: intracoronary ultrasound and pressure measurements. Br Heart J.

[11] Ishii T, Asuwa N, Masuda S, Ishikawa Y (1998). The effects of a myocardial bridge on coronary atherosclerosis and ischaemia. J Pathol.

[12] Abdil-Rauf Z, Odeh M, Blinder J, Rosenschein U, Barmeir E (2007). Myocardial bridge: evaluation on MDCT. AJR Am J Roentgenol.

[13] Wan L, Wu Q (2005). Myocardial bridge, surgery or stenting?. Interact Cardiovasc Thorac Surg.

[14] Chai PJ (2018). Surgical intervention for myocardial bridges: to do or not do to? That is the question. J Thorac Cardiovasc Surg.

[15] Attaran S, Moscarelli M, Athanasiou T, Anderson J (2013). Is coronary artery bypass grafting an acceptable alternative to myotomy for the treatment of myocardial bridging?. Interact Cardiovasc Thorac Surg.

[16] Mok S, Majdalany D, Pettersson G (2019). Extensive unroofing of myocardial bridge: a case report and literature review. SAGE Open Med Case Rep.

[17] (2019). Myocardial Bridging of the Coronary Arteries. http://www.uptodate.com/contents/myocardial-bridging-of-the-coronary-arteries.

